# Neural systems and hormones mediating attraction to infant and child faces

**DOI:** 10.3389/fpsyg.2015.00970

**Published:** 2015-07-17

**Authors:** Lizhu Luo, Xiaole Ma, Xiaoxiao Zheng, Weihua Zhao, Lei Xu, Benjamin Becker, Keith M. Kendrick

**Affiliations:** Key Laboratory for NeuroInformation of Ministry of Education, Center for Information in BioMedicine, University of Electronic Science and Technology of ChinaChengdu, China

**Keywords:** baby schema, infant face, neural circuitry, parental behavior, hormones

## Abstract

We find infant faces highly attractive as a result of specific features which Konrad Lorenz termed “Kindchenschema” or “baby schema,” and this is considered to be an important adaptive trait for promoting protective and caregiving behaviors in adults, thereby increasing the chances of infant survival. This review first examines the behavioral support for this effect and physical and behavioral factors which can influence it. It then provides details of the increasing number of neuroimaging and electrophysiological studies investigating the neural circuitry underlying this baby schema effect in parents and non-parents of both sexes. Next it considers potential hormonal contributions to the baby schema effect in both sexes and the neural effects associated with reduced responses to infant cues in post-partum depression, anxiety and drug taking. Overall the findings reviewed reveal a very extensive neural circuitry involved in our perception of cuteness in infant faces, with enhanced activation compared to adult faces being found in brain regions involved in face perception, attention, emotion, empathy, memory, reward and attachment, theory of mind and also control of motor responses. Both mothers and fathers also show evidence for enhanced responses in these same neural systems when viewing their own as opposed to another child. Furthermore, responses to infant cues in many of these neural systems are reduced in mothers with post-partum depression or anxiety or have taken addictive drugs throughout pregnancy. In general reproductively active women tend to rate infant faces as cuter than men, which may reflect both heightened attention to relevant cues and a stronger activation in their brain reward circuitry. Perception of infant cuteness may also be influenced by reproductive hormones with the hypothalamic neuropeptide oxytocin being most strongly associated to date with increased attention and attraction to infant cues in both sexes.

## Introduction

The faces of both infants and young children are potent cross-cultural emotive stimuli that adults find both very cute and highly likeable and evoke feelings of protectiveness and care which thereby serve to aid survival of these vulnerable individuals (Brosch et al., 2007; Luo et al., 2011, 2014; Proverbio et al., 2011a; Borgi et al., 2014). Konrad Lorenz (1943) has defined this as the so-called “baby schema” or “Kindchenschema” effect.

Baby schema are considered to be a set of prominent infantile facial physical features, including large head, round face, high and protruding forehead, big eyes, small nose, small mouth, etc., that evoke rapid cognitive, affective and behavioral responses in adults. This may serve as an innate releasing mechanism, a fundamental social instinct which serves to initiate and maintain a parent/carer-infant relationship, particularly during the period of early development when a child is unable to care for itself and is therefore highly vulnerable (Parsons et al., 2010). As such this mechanism helps infant individuals develop secure and cooperative relationships, improves their adaptation to the society and thereby enhances offspring/species survival (Darwin, 1872; Parsons et al., 2010). A number of studies have also reported correlations between perceived cuteness of infant faces and health (Yamamoto et al., 2009), including across cultures (Volk and Quinsey, 2002; Volk, 2009; Golle et al., 2015). Since adult facial attractiveness has often been associated with having “good genes” (Weeden and Sabini, 2005; Rhodes, 2006), it is possible that prominent baby schema may also signal that infants are genetically healthy. Thus, as has been proposed by Golle et al. (2015), cute babies by being perceived as more healthy may promote greater protective and nurturing responses in carers, thereby serving to strengthen a community gene pool.

It is well established that viewing infant and child faces produces positive effects in adult observers both within and across different cultures in terms of automatically and rapidly capturing their attention (Brosch et al., 2007, 2008; Proverbio et al., 2011a), evoking smiling (Schleidt et al., 1980), protective reactions (Alley, 1983), close approach and exaggerated greeting responses (Eibl-Eibesfeldt, 1989). Studies demonstrating increased attentional allocation to infant faces have used a wide variety of paradigms, including a “key-press,” or “wanting” task (as in Parsons et al., 2014), an attentional capture task (as in Thompson-Booth et al., 2014), eye-tracking (as in Borgi et al., 2014), and the dot-probe task (Brosch et al., 2007).

Adult Caucasian observers presented with a choice between a cute and less cute infant face exhibit a preference for giving a toy to, or adopting, the cute infant irrespective of whether the infant is Caucasian or African, or even a dog puppy (Golle et al., 2015). A recent study has also shown that faces with baby schema not only evoke positive emotions and caring behaviors in adults but can even evoke enhanced ratings of cuteness in young children aged 3–6 years old (Borgi et al., 2014). Overall, children whose faces display strong baby schema features are perceived as being cuter, friendlier, healthier, more attractive, trustworthy, and adoptable (Karraker and Stern, 1990; Ritter et al., 1991; Chin et al., 2006; Glocker et al., 2009a; Little, 2012; Golle et al., 2015). A recent study using an infrared thermography technique revealed that in both Italian and Japanese subjects facial temperature, a physiological index of arousal, was significantly increased during perception of infant faces from both in-group and out-group cultures, whereas with adult faces this was only the case for those from in-group members (Esposito et al., 2014). Finally, in terms of a potential direct influence of baby schema on parental behavior, a study has reported that mothers with cute infants showed greater affection and playfulness toward them than did mothers with infants who were less cute (Langlois et al., 1995).

While it was originally thought that the effects of baby schema on perceivers were limited to young infants of <1 year of age, it is now clear that they can also extend to faces of children of up to 4.5 years of age (Luo et al., 2011). Indeed, even adult faces with “babyish,” immature features are considered more attractive, lovable, warm, submissive, physically weak and naive (McArthur and Apatow, 1984; Berry and McArthur, 1986). Imagined interactions with individuals exhibiting these babyish features are also associated with an increased feeling of social belonging in observers (Sacco et al., 2014). Thus findings suggest a generalization of attractiveness of infantile face features across both children and adults (Zebrowitz et al., 2009). The baby schema effect also appears to extend to our perception of cuteness in the young of other species (Golle et al., 2013, 2015; Lehmann et al., 2013; Borgi et al., 2014), and exhibits high level perceptual “after effects” similar to observations for a number of key features in adult faces (i.e., prolonged perception of cute infant faces lowers attraction ratings given to less cute ones presented subsequently and vice versa- Golle et al., 2013).

The baby schema effect is not just dependent upon the presence of the relevant salient physical facial features (Glocker et al., 2009b; Komori and Nittono, 2013) and can, for example, be weakened by the presence of some form of facial disfigurement (Baeken et al., 2010a). A study has also investigated the influence of temperament on attractiveness of neutral expression infant faces by pairing them with happy or sad facial expressions and equivalent vocalizations. Infant faces paired with mostly happy faces and vocalizations are perceived as even cuter and adult observers are prepared to exert greater effort to view them. On the other hand observers of infant faces paired with mostly sad face expressions and vocalizations don't rate them as cuter and are less prepared to make an effort to view them (Parsons et al., 2014). Thus an infant's temperament can enhance or decrease their perceived attractiveness.

The baby schema effect can also be influenced by an observer's social experience. Adults raised together with siblings like infant and child faces more than those who were not, and the smaller the mean age difference between them and their siblings the greater this effect is (Luo et al., 2014). Similarly, Caucasian children with older siblings had a higher accuracy in recognizing unfamiliar Caucasian child faces relative to Asian ones, while those without them didn't show any such recognition difference between the faces (Macchi Cassia et al., 2014). Parental experience is also influential since mothers in an attention capture paradigm showed longer reaction times to infant vs. adult faces, suggesting that they were more attentive toward infant faces. Reaction times were also negatively correlated with self-reported parental distress in mothers (Thompson-Booth et al., 2014). However, another study did not find significant reaction time differences in mothers and non-mothers in judging infant face expressions (Nishitani et al., 2011).

Personality of the observer can also influence the effects of baby schema. Individuals with higher levels of trait empathy, interpersonal closeness and needing to belong rate infant faces more positively, while personality attributes such as narcissism and insecure attachment have no influence (Lehmann et al., 2013). Finally, degree of resemblance of a child's face to that of the observer has also been reported to increase their perceived attractiveness in both men and women (DeBruine, 2004).

In recent years there has been increasing interest in establishing the neural (Nitschke et al., 2004; Glocker et al., 2009b; Malak et al., 2015) and hormonal influences (Bhandari et al., 2014; Hahn et al., 2014) underlying attraction to baby schema in infant and child faces. There is also an increasing focus on understanding changes in the early post-partum period which are crucial for the formation of mother-infant bonds and factors which may contribute to the development of post-partum depression. For example, studies suggest that an appraisal bias might underlie some of the difficulties mothers with post-partum depression have in responding to cues from their own infant's signals, since they are more likely to rate less cute infant faces negatively (Stein et al., 2010), and rate even average ones more negatively than other mothers (Gil et al., 2011). Substance abuse has also been associated with altered behavioral and neural responses to infant cues (Landi et al., 2011). In the present review we will therefore summarize and discuss the various neural and hormonal influences on infant and child facial processing and attraction established in healthy observers and also clinical research findings in disorders where there is some impairment involved.

## Neural responses to infant faces: baby schema

As would be expected, emotive and salient social stimuli such as infant faces provoke widespread activation in brain systems involved in face perception, attention, emotion, empathy, memory, reward and attachment, theory of mind and also control of motor responses (see Table 1). The question of whether there is something special about brain processing of infant faces is therefore difficult to address given the involvement of so many different systems. Very few studies have investigated the influence of baby schema *per se* but have simply investigated neural responses to infant or child faces either alone or in comparison to adult human faces or young and adult faces from other species (Barrett et al., 2012; Caria et al., 2012; Stoeckel et al., 2014). The experimental protocols used in these studies are summarized in Table 1 where it can be seen that the majority use a simple face viewing paradigm, although some have also used a one-back working memory task with faces, an oddball task design, face expression judgments or an affect rating task. Only Glocker and her colleagues have objectively quantified and parametrically manipulated the impact of baby schema content on patterns of brain activations (Glocker et al., 2009a,b). Thus, conclusions drawn from the present review are primarily based on the majority of findings reporting differences in neural responses between viewing infant as opposed to adult faces, or between viewing the faces of own as opposed to other infants.

**Table 1:** **Neural responses to infant and child faces**.

**Study**	**Participants (Age; N: M/F)**	**Face age**	**Stimuli**	**Design**	**Face duration**	**Paradigm**	**Contrasts**	**Findings: activated brain regions (Brodmann area)**	**Corrections**
**FUNCTIONAL MAGNETIC RESONANCE IMAGING (fMRI) STUDIES**									
Bartels and Zeki, 2004	Healthy mothers (27–49 years; 20:0/20)	9 months to 6 years	Child face pictures	Block	2.5 s	View	Own > acquainted child	Middle INS (14)^*^ Dorsal ACC (24)^*^ Ventral ACC (24) dorsal CAU^*^ medial PUT/GP^*^ Lateral THA R SNi^*^ Lateral OFC	*p* < 0.001 (uncorrected)^*^ p <0.05 (SVC corrected)
							Acquainted > own child	STS (39, 40)^*^ PCC (29, 30)^*^ Medial PCU (7/31)^*^ MTG (21)^*^ AMY^*^	
							(Own > acquainted child) > (loved partner > friends)	Lateral OFC	
Leibenluft et al., 2004	Healthy mothers (20–40 years; 7:0/7)	5–12 years	Pictures (friend, unfamiliar child, and unfamiliar adult)	Event	1.5 s	One-back memory	Own > familiar child	L ACC (32) L preCG SFG (6) L pSTS (39) R STG (22) MTG (21) R PCC (23) R PCU (31) L SMG (40) R AMY L INS THA GP R PUT Cerebellum	*p* < 0.025 (uncorrected)
							Familiar > unfamiliar children	R ACC (32) R SFG (8) MFG (46/9/47) L posterior OFC (11) L pSTS (39) R MTG (21) L PCC-PCU (39) SMG (40) IPS (40) R MOG (18) R FFG (37) L AMY INS THA L CAU Cerebellum	
							Unfamiliar children > unfamiliar adults	L posterior OFC (11) R pSTS (39) L MTG (21) L PCC-PCU (23/31) R SMG (40) L FFG (37) L INS R THA	
Nitschke et al., 2004	Healthy mothers (-; 6: 0/6)	3–5 months	Happy faces neutral images	30-s block design	6 s	Mood rating	Own infants > baseline (blank screen)	Lateral OFC (all 6 subjects	*p* < 0.05 (uncorrected)
							Unfamiliar infants > baseline	Lateral OFC (only 2 subjects)	^*^ *p* < 0.001 (corrected)
							Own > unfamiliar infants	Lateral OFC (11/47)^*^ Cerebellum^*^	
							Unfamiliar > own infants	ATC (20/21) ^*^	
Ranote et al., 2004	Healthy mothers (19–35 years; 10: 0/10)	4–8 months	Video stimuli	Block	40s	View	Infants > neutral	Cerebellum MOG (19) L MTG (21/37/39)	*p* < 0.001 (uncorrected)
								STG (38) postCG (4)	^*^p < 0.05 (SVC corrected)
							Own > unknown infants	R MOG (19) L AMY^*^	
							Unknown > own infants	lateral OFC (47) R MTG (21) L PCU (18) L postCG (7)	
Noriuchi et al., 2008	Healthy mothers (31.1 ± 2.2 years; 13:0/13)	16.5 ± 3.8 months	Video stimuli (smile, cry)	Block	32 s	View	Own > other infant	OFC (47)^*^ R MTG (39) R anterior INS^*^ L PCC (30)	*p* < 0.001, (uncorrected)
								L THA^*^ L PUT	^*^ *p* < 0.05 (FWE corrected)
							Other > own infant	R STG (22) L IPS (40) L PCU (7) L FFG (36) L HIPP (37) PUT^*^	
							Own infant: separation > play situation	SL CAU^*^ IPS (40) L PCC (31) L SNi^*^ R MTG (21) L OFC (47) PCU (7/31) PREC (4) R ACC (32) SFG (6) STG (22/39/42) STS (21/22) THA^*^	
							own infant: play > separation situation	R MTG (22/41) SMG preCG (4) L hypothalamus^*^	
Strathearn et al., 2008	Healthy first-time mothers (20–42 years; 28:0/28)	5–10 months	Baby face pictures (happy, neutral, sad)	Event	2 s	View	Own > unknown baby	L lateral OFC (47) R preCG (4) postCG MTG (21/38) STG (22/21) PUT dorsal CAU THA Lateral superior AMY HIPP (36) INS ACC (24/32) MCC (24) PCC (31/17) VTA SNi Cerebellum	*p* < 0.05 (FDR corrected)
							Own > unknown happy infant faces	PUT L SNi R THA L AMY	
							Own > unknown neutral infant faces	R PUT R medial dorsal/ventrolateral THA L dorsal PUT	
Glocker et al., 2009b	Healthy mothers (20–28 year; 16:0/16)	7–13 months	Baby schema pictures (high, low, original)	Event	3 s	Cuteness rating	Infant faces > cross	THA INS (13) SFG (6) Cerebellum PCU (7) postCG (2) preCG (6)	*p* < 0.05 (FWE corrected)
							High baby schema > unmanipulated & low baby schema	L ACC L PCU L FFG R NAcc	
Caria et al., 2012	Healthy adult non-parents (28.06 ± 5.66 years; 16:7/9)	–	Infant and adult human and animal face pictures	Event	4 s	View	Infant > adult	SMA (6) FFG (37/19) preCG (6) MCC (31/24) L anterior INS (48) THA	*p* < 0.01 (FWE corrected)
Montoya et al., 2012	Healthy nulliparous women (19–29 years; 17:0/17)	5–10 months	Unknown infant face pictures (happy, sad, neutral)	Event	1 s	One-back memory	Happy > neutral	L OFC (11)	*p* < 0.05 (FWE corrected)
							Neutral > happy	R STG (22) L INS (13) L preCG (4)	
							Sad > neutral	R PCU (31) R MTG (39) L preCG (6) R SFG (10) R OFC (11)	
							Neutral > sad	L INS (13) R STG	
							Sad > happy	R FFG (19) R STG (39) R preCG (6) R FFG (19)	
Baeken et al., 2010a	Healthy females (26.6 ± 6.9 years; 20:0/20)	5.5 ± 4 months	Baby face pictures (neutral, positive, negative)	Block	3.6 s	view	Positive > neutral	FFG (37/19)	*p* < 0.001 (uncorrected)
							Negative > neutral	FFG (18/37/19)	
Barrett et al., 2012	Healthy mothers (25–35 years; 22:0/22)	~3 months	Baby face pictures (positive, negative)	Block	3 s	Affect-rating task (ART)	Positive: own > unfamiliar	Cerebellum STG (38) MTG (21) AMY TH	*p* < 0.001 (uncorrected)
							Negative: own > unfamiliar	PUT postCG(3) STG (38) Cerebellum	
Lenzi et al., 2009	Healthy mothers (23–42 years; 16:0/16)	6–12 months	Baby face pictures (joy, distress, ambiguous, neutral)	Block	2 s	View/imitate	Imitation: emotive > neutral	STS AMY	*p* < 0.05 (FWE, SVC corrected)
							Observation: emotive > neutral	IPS INS AMY	
							Observation: own > other child	IPS Anterior INS STS	
Strathearn et al., 2009	Primiparous mothers (N/A; 30:0/30)	~11 months	Infant face pictures (happy, neutral, sad)	Event	2 s	View	Own > unknown (secure > insecure)	Hypothalamus	*p* = 0.0001
							Own > unknown (insecure > secure)	anterior INS (13)	
							Own happy faces: secure > insecure	OFC (10/45/46)	
							Own happy faces: insecure > secure	dlPFC (9/46) SCG	
							Own sad faces: secure > insecure	lPFC (9) Ventral Striatum / NAcc	
							Own sad faces: insecure > secure	anterior INS (13)	
Zebrowitz et al., 2009	Healthy subjects (21–36 years;17:8/9)	5–9 months	Babies, babyfaced/maturefaced men pictures	Block	200 ms	View	Baby faces > mature faced adults	AMY	*p* < 0.05
Landi et al., 2011	Substance-using mothers (26:0/26); non-using mothers (28:0/28); 17–42 years;	5–10 months	Infant faces (happy, neutral, sad)	Event	1 s	One-back memory task	Happy infant faces: non-using>substance-using mothers	R postCG L SFG R HIPP/ parahippocampus L cerebellum	*p* < 0.05 (corrected)
							Happy infant faces: substance-using>non-using mothers	L. posterior parahippocampal gyrus	
							Sad infant faces: non-using>substance-using mothers	medial OFC MTG/STG PCC R AMY Parahippocampal gyrus	
Kuo et al., 2012	Healthy fathers (28–44 years; 10:10/0)	8–19 weeks	Videos stimuli (neutral or slight positive)	Block	15 s	View	Own > other infant	R SFG CAU R OFC	*p* < 0.05 (FDR corrected)
							Other > own infant	FFG	
Laurent and Ablow, 2013	Primiparous mothers (24.1 ± 4.1 years; 22:0/22) Half with depressive symptoms	15–18 months	Infant face pictures (joy, distress)	Block	6 s	View	Own > other infant joy faces (mothers with lower current self-reported depressive symptoms)	R INS L inferior OFC (11)	*p* < 0.05 (FDR corrected)
							Own > other infant distress faces (non-depressed > depressed mothers)	L dorsal ACC (32)	
							Own infant joy > distress faces (mothers with lower current self-reported depressive symptoms)	L INS- PUT L dorsal ACC-SMA (24/6) L SMG (40)	
Strathearn and Kim, 2013	Healthy primiparous mothers (28.5 ± 0.8 years; 39:0/39)	6.8 ± 0.3 months	Infant face pictures (happy, neutral, sad)	Event	2 s	View	Own > unknown happy infant faces	R preCG (4) R SFG (6) L postCG (3) R STG (38) R PUT L AMY / Dorsal striatum R Dorsal CAU R Dorsal PUT SNi/VTA	*p* < 0.005 (FDR corrected)
Stoeckel et al., 2014	Healthy mothers (22–45 years; 14:0/14)	2–10 years	Dog and child pictures	Block	4s	View	Own > unfamiliar child	FFG (37)^*^ PUT^*^ SNi/VTA THA^*^	*p* < 0.05 (FWE corrected)
							Own child > fixation	AMY^*^ FFG^*^ R HIPP^*^ Medial OFC^*^ PUT^*^ SNi/VTA^*^ THA^*^	^*^p < 0.01 (FWE corrected)
Wan et al., 2014	Healthy mothers (20–43 years; 20:0/20)	4–9 months	Own and unfamiliar infant videos with neutral to mildly positive affect, and emotionally neutral stimuli (moving traffic)	Block	30 s	View (Infant video activation paradigm)	Infants > moving traffic	L Cerebellum (19)^*^ R MTG (39)^*^ R FFG (37)^*^	*p* < 0.001 (uncorrected)
							Own > unknown infant	Cerebellum PCU (7) R STG (38)	^*^p < 0.05 (FDR corrected)
								R IPS (40) R postCG (1) preCG (4/6) L AMY^**^	^*^p < 0.05 (FDR corrected)
							Unknown > own infant	MTG (21); Cerebellum	
							Own > unknown infant (correlates with maternal nondirectiveness)	FFG (18) PUT	
							Own > unknown infant (correlates with infant interactive behavior)	preCG (4) THA	
							Own > unknown infant (correlates with mothers' perceived warmth of her infant)	PCU (7) INS (13)	
**MAGNETOENCEPHALOGRAPHY (MEG) STUDIES**									
Kringelbach et al., 2008	Healthy subjects (29.5 years; 12:5/7)	3–12 months	Positive, negative, neutral	Block	300 ms	View	Specially found on infant faces, not adult faces	L mOFC: 10–15 Hz, 130 ms R FFG: 20–25 Hz, 165 ms	
**EVENT-RELATED POTENTIALS (ERP) ELECTROENCEPHALOGRAPH (EEG) STUDIES**									
Doi and Shinohara, 2012	Healthy mothers (33.7 ± 4.3 years; 16: 0/16)	*M* = 5.6 ± 0.8 years	Children face pictures (eyes closed, gaze straight, gaze averted)	Block	1000 ms	Oddball paradigm (Respond to a face with its eyes open)	Straight > averted gazes	Own child (but not an unfamiliar child): N170	
							Unfamiliar >own child	Straight gaze: P3	
Proverbio et al., 2011b	Healthy subjects (19–27 years; 40:20/20)	N/A	Adults, children, infants, objects, landscape pictures	Event	800 ms	An implicit task with key press only to landscape pictures	Infants > adults	N1	
							Infants > children	Female: anterior N2	
							Children > adults	Male: anterior N2	
Weisman et al., 2012c	Healthy parents (19–33 years; 24:13/11) Healthy lovers (19–33 years; 19:10/9) Healthy singles (19–33 years; 22:12/10)	6 months	Infant face pictures (neutral); standard landscape stimuli	Event	300 ms	Oddball paradigm (Press a key whenever an infant's face appeared on screen)	Unfamiliar infant faces: parents & lovers > singles (subjects)	Occipital–lateral (N170); central–frontal (P3a) sites;	
							Own > unfamiliar	Parietal-distributed P300 component	
Esposito et al., 2015	Healthy primiparous mothers (32.06 ± 4.66 years; 21:0/21)	3–6 months	Infant faces pictures (neutral)	Event	500 ms	View	Own > unfamiliar	Midline occipital (Oz) cluster;	
							Unfamiliar > own	Right temporal (Tr) cluster; left temporal (Tl) cluster;	
Malak et al., 2015	Mothers (28 ± 5.7 years; 47:0/47)	1–23 months	Unfamiliar infant faces pictures (neutral, distressed)	Event	1500 ms	View	Distressed > neutral	↑ LPP - The degree of late Positive potential (LPP) amplitude elicited by neutral infant faces was positively correlated with state anxiety.	
**NEAR-INFRARED SPECTROSCOPY (NIRS) STUDIES**									
Minagawa-Kawai et al., 2009	Healthy mothers (28–42 years; 18:0/18)	9–13 months	Video stimuli (neutral, smile)	Block	30 s	View	Own > unfamiliar	OFC	
Nishitani et al., 2011	14 healthy mothers (20–42 years; 14:0/14); 14 healthy non-mothers (20–42 years; 14:0/14)	9–36 months	Infant and adult face pictures (happy, angry, sad, fearful, surprised, neutral)	Event	N/A	Expression judgment	N/A	↑ R PFC activity (discriminating infant facial emotions but not adult facial emotions)	

*N, numbers of subjects; M, male; F, female; L, left; R, right. Abbreviations of brain regions: see Table 2*.

**Figure 1:**
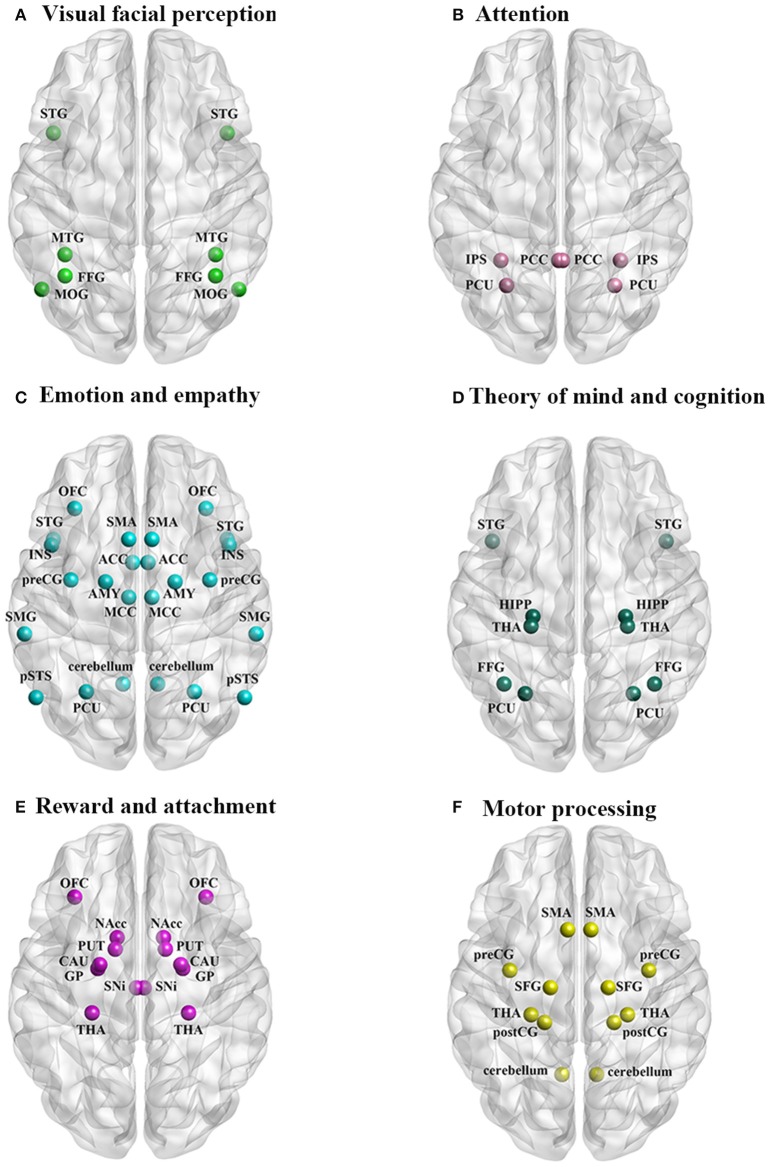
**The neural circuitry activated by perception of infant faces in parents and non-parents of both sexes associated with: (A) visual facial perception, (B) attention, (C) emotion and empathy, (D) theory of mind and cognition, (E) reward and attachment, and (F) motor processing**.

In general neuroimaging studies, primarily using functional magnetic resonance imaging (fMRI), have shown that there is a degree of overlap in neural processing of infant and adult faces since both activate primary visual processing areas as well as those more specifically associated with face perception, such as the fusiform face area (FFA) (for abbreviations of brain regions see Table 2) (Kringelbach et al., 2008; Glocker et al., 2009b; Baeken et al., 2010a; Stoeckel et al., 2014). However, infant faces generally elicit more rapid responses and greater activity changes in these brain areas and additionally recruit other regions (Parsons et al., 2010; Caria et al., 2012; Hahn et al., 2014). In regions showing activation in response to both adult and infant faces stronger responses to infant/child faces have been reported in the fusiform gyrus [FFG—Brodmann area (BA) 41/37/19] (Leibenluft et al., 2004; Kringelbach et al., 2008; Caria et al., 2012; Stoeckel et al., 2014), middle occipital gyrus (MOG—BA 19/37) (Ranote et al., 2004; Caria et al., 2012), medial temporal gyrus (MTG—BA 21/37/39) (Leibenluft et al., 2004; Ranote et al., 2004; Caria et al., 2012) and superior temporal gyrus (STG—BA 38) (Ranote et al., 2004; Stoeckel et al., 2014) (Figure 1A). Of these regions, the right FFG in particular is of key importance to face processing (see Leopold and Rhodes, 2010) and may play a vital role in encoding baby schema facial features (Hoffman and Haxby, 2000; Glocker et al., 2009b; Stoeckel et al., 2014). These visual cortical areas may serve as an entry node to forward the processed baby face information to other brain regions associated with attention, emotion and memory for further processing and control of behavioral responses (Glocker et al., 2009b).

**Table 2:** **The abbreviations and full names of activated brain regions**.

**Abbreviations**	**Full names**
ACC	Anterior Cingulate Cortex
AMY	Amygdala
ATC	Anterior Temporal Cortex
ATP	Anterior Temporal Pole
APC	Anterior Paracingulate Cortex
CAU	Caudate
dlPFC	Dorsal Lateral Prefrontal Cortex
FFG	Fusiform Gyrus
GP	Globus Pallidus
GM	Gray Matter
HIPP	Hippocampus
IFG	Inferior Frontal Gyrus
INS	Insula
IPL	Inferior Parietal Lobule
IPS	Intraparietal Sulcus
lPFC	Lateral Prefrontal Cortex
MCC	Medial Cingulate Cortex
MFG	Medial Frontal Gyrus
MOG	Middle Occipital Gyrus
MTG	Middle Temporal Gyrus
mPFC	Medial Prefrontal Cortex
MTG	Medial Temporal Gyrus
NAcc	Nucleus Accumbens
OFC	Orbitofrontal Cortex
PAG	Periaqueductal Gray
PCC	Posterior Cingulate Cortex
PCU	Precuneus
PFC	Prefrontal Cortex
PHG	Parahippocampal Gyrus
preCG	Precentral Gyrus
postCG	Postcentral Gyrus
pSTS	Posterior Superior Temporal Sulcus
PUT	Putamen
SCG	Subcallosal Gyrus
SFG	Superior Frontal Gyrus
SMA	Supplementary Motor Area
SMG	Supramarginal Gyrus
SNi	Substantia Nigra
SPL	Superior Parietal Lobe
STG	Superior Temporal Gyrus
TC	Temporal Cortex
THA	Thalamus
VTA	Ventral Tegmental Area

Infant and child faces also enhance attention and this is reflected in stronger activation of parietal areas involved both bottom up and top down processing of attention orientation (Shomstein, 2012) including the intraparietal sulcus (IPS—BA 19/7) (Leibenluft et al., 2004), precuneus (PCU—BA 7/31) (Leibenluft et al., 2004; Glocker et al., 2009b), and posterior cingulate cortex (PCC—BA 23) (Leibenluft et al., 2004) (Figure 1B). Greater activation of these parietal regions selectively help allocate more automatic and cognitive attentional resources to faces with baby schema features resulting in an attentional bias toward the infant faces (Brosch et al., 2007; Glocker et al., 2009b; Caria et al., 2012). An evoked-related potential (ERP) study has also reported stronger activation in response to neutral expression faces of unfamiliar infants at central–frontal (P3a) and occipital–lateral (N170) sites providing further support for increased attention toward infant cues (Weisman et al., 2012c).

There is also evidence that infant faces elicit strong activation in brain regions associated with core aspects of emotion processing (see Lindquist et al., 2012) including the anterior cingulate cortex (ACC—BA 33/24) (Glocker et al., 2009b) and medial cingulate cortex (MCC—BA 31/23/24) (Caria et al., 2012), insula (INS—BA 48/47/13) (Leibenluft et al., 2004; Glocker et al., 2009b; Caria et al., 2012; Stoeckel et al., 2014), amygdala (AMY; Lenzi et al., 2009; Barrett et al., 2012; Wan et al., 2014), orbitofrontal cortex (OFC—BA 11/47) (Leibenluft et al., 2004; Nitschke et al., 2004; Kringelbach et al., 2008; Minagawa-Kawai et al., 2009; Kuo et al., 2012; Wan et al., 2014) (Figure 1C). The OFC also plays an important role in learning the emotional value of information and tracking changing emotions (Goodkind et al., 2012), as well as with judgments of pleasantness (Bartels and Zeki, 2004). Thus, the left OFC (BA 11) is more strongly activated by happy vs. neutral infant faces while the right OFC (BA 11) to sad vs. neutral faces (Montoya et al., 2012; Stoeckel et al., 2014). Finally, a repetitive transcranial magnetic stimulation (rTMS) study has found that a single high frequency session with stimulation applied to the left dorsolateral prefrontal cortex enhanced processing of positive emotions on baby faces and reduced that for negative emotions (Baeken et al., 2010b). Thus infant faces elicit stronger responses in emotional brain circuitry involved with processing both valence and arousal. This would suggest that overall infant faces evoke both stronger arousal and enhanced responses to both positive and negative cues from the infant. The OFC and dorsolateral prefrontal cortex in particular appear to play a key role in mediating differential responses to positive and negative valence infant faces.

Many of the core brain regions engaged in emotion processing are also involved with core processing of empathy, notably the MCC, INS, and OFC (Fan et al., 2011), and other regions in the empathy network also show strong activation in response to infant faces, notably the posterior superior temporal sulcus (pSTS; Leibenluft et al., 2004), supplementary motor area (SMA—BA 6) and precentral gyrus (preCG; Caria et al., 2012), STG (Ranote et al., 2004; Stoeckel et al., 2014), PCU (Leibenluft et al., 2004; Glocker et al., 2009b), right supramarginal gyrus (SMG—BA 40) (Leibenluft et al., 2004), and also cerebellum (Ranote et al., 2004; Glocker et al., 2009b) (Figure 1C). Overall this pattern of enhanced activity in empathy processing regions suggests that it may contribute to better identification of emotions being expressed (cognitive empathy) and enhanced empathic feelings toward the infant (affective empathy). Furthermore, increased activity in preCG and SMA might lead to increased motor empathy in terms of mimicry of facial expressions. Interestingly, a recent paper has shown that 18 month old infants exposed to a higher level of mimicry by their mothers exhibited increased prosocial behavior (Carpenter et al., 2013).

Enhanced activity changes in response to infant faces in the pSTS, PCU and PCC (Leibenluft et al., 2004; Glocker et al., 2009b) may also reflect an impact on core processes for theory of mind (Schurz et al., 2014). Theory of mind refers to the capacity to attribute mental states to oneself or others, and to predict and account for other people's behavior based upon understanding of their intentions and mental states (Premack and Woodruff, 1978; Leibenluft et al., 2004). Greater activation in these areas may therefore help adults utilize previous memorized experience and all the skills they have to better understand and respond toward the infant and successfully manage their social communication and relationship. Linked to this there is also evidence that infant faces evoke greater activation in regions associated with episodic memory including the hippocampus (HIPP; Stoeckel et al., 2014), PCU (Leibenluft et al., 2004; Glocker et al., 2009b) and thalamus (THA; Leibenluft et al., 2004; Caria et al., 2012; Stoeckel et al., 2014) (Figure 1D). Enhanced activation in FFG (BA 41/37/19) (Leibenluft et al., 2004; Kringelbach et al., 2008; Caria et al., 2012; Stoeckel et al., 2014) and STG (Ranote et al., 2004; Stoeckel et al., 2014) may additionally contribute through their role in social cognition.

A number of studies have addressed the question of whether infant faces are particularly rewarding. Findings have consistently shown that infant faces appear to evoke greater activation of regions involved in reward and attachment, including the OFC (Leibenluft et al., 2004; Nitschke et al., 2004; Kringelbach et al., 2008; Minagawa-Kawai et al., 2009; Kuo et al., 2012; Stoeckel et al., 2014; Wan et al., 2014), substantia nigra/ventral tegmental area (SNi/VTA; Stoeckel et al., 2014), nucleus accumbens (NAcc)/ventral striatum (Stoeckel et al., 2014), caudate (CAU; Glocker et al., 2009b; Kuo et al., 2012; Stoeckel et al., 2014), putamen (PUT) (Glocker et al., 2009b; Stoeckel et al., 2014), globus pallidus (GP) (Glocker et al., 2009b; Stoeckel et al., 2014) and the THA (Leibenluft et al., 2004; Caria et al., 2012; Stoeckel et al., 2014) (Figure 1E). Although the OFC is also involved in emotion processing, its contribution to enhanced rewarding properties of infant faces has been particularly emphasized. The NAcc and VTA are key interconnected regions in the dopaminergic brain reward system (Bourdy and Barrot, 2012) which are selectively activated during both overt perception and mental imagery of rewarding and reinforcing stimuli, especially pleasant and emotionally arousing ones such as infant faces (Costa et al., 2010). Furthermore, lesions in NAcc have been reported to impair the baby schema effect (Numan, 2007). The NAcc and VTA are important for reward-mediated attachment and affiliation (Stoeckel et al., 2014) and also appear to be important targets whereby prosocial hormones such as oxytocin and vasopressin exert their effects on social reward (Scheele et al., 2013).

One of the most notable effects of infant faces is that they evoke stronger activation in brain motor areas than adult faces, including the SMA (BA 6) (Caria et al., 2012), precentral gyrus (preCG—BA 6) (Caria et al., 2012; Glocker et al., 2009b), postcentral gyrus (postCG—BA 2/3/4) (Glocker et al., 2009b; Ranote et al., 2004), superior frontal gyrus (SFG—BA 6) (Glocker et al., 2009b; Caria et al., 2012; Kuo et al., 2012; Wan et al., 2014), THA (Leibenluft et al., 2004; Caria et al., 2012; Stoeckel et al., 2014) and cerebellum (Ranote et al., 2004; Glocker et al., 2009b) (Figure 1F). These interconnected areas form the core motor circuit for preparation and planning and execution of intentional movements and speech (Goldberg, 1985; Caria et al., 2012). The SFG contains a number of different sub-regions with different patterns of connectivity with regions involved in motor control, working memory and attention and self-awareness (Li et al., 2013), and may play an important role in integrating perception and action (Goldberg et al., 2006). Overall the extensive pattern of activation in motor control circuitry seen in response to infant faces may be particularly important in mediating unconscious, intuitive and virtually unavoidable patterns of approach behavior in adults to protect infants and promote both physical and language interactions with them (Ackermann and Ziegler, 2010; Caria et al., 2012). As already discussed above in relation to motor empathy, adult observers may also show a greater propensity to mimic infant face expressions thereby aiding infant prosocial development (Carpenter et al., 2013).

Only a small number of studies have specifically investigated the impact of the intensity of baby schema features in infant faces either by comparing responses to the faces of infants (high baby schema) and children (low baby schema) or through direct manipulation of baby schema features in specific face stimuli. From these studies there is evidence that intensity of baby schema does influence neural responses in visual and face processing regions as well as in regions controlling emotional responses, attention and memory, theory of mind and reward. An ERP study found that N1 amplitude localized in the FFG was increased in response to infant faces compared to adult, although not child faces, in both males and females. On the other hand N2 amplitude localized in the medial occipital cortex and FFG, uncus and medial OFC was increased in response to infant compared with child and adult faces, although only in females (Proverbio et al., 2011b). An fMRI study which specifically manipulated the intensity of baby features found that infant faces with high baby schema features, and which were rated to be very cute, produced stronger activation than ones with low baby schema features in the right NAcc, left ACC (BA 24), left PCU (BA 7), and left FFG (Glocker et al., 2009b). Interestingly, another fMRI study showed that adult faces with a baby-faced appearance and infant faces evoked greater activation in the same brain regions (AMY and FFA) than more mature looking adult faces, and also stimulated greater functional connectivity between them (Zebrowitz et al., 2009). However, it should be noted that the enhanced impact of baby faces on AMY and OFC responses is disrupted if the infant face has physical anomalies, which disrupt the baby schema (Baeken et al., 2010a; Parsons et al., 2013). Overall therefore evidence from these studies indicates that infant faces with high baby schema are more likely to evoke greater responses in brain regions processing faces, attention, emotion and positive reward than those with low baby schema.

## Specific neural responses to own infant faces: parental love

In addition to the general impact of baby schema on neural processing discussed above there is widespread evidence for further enhanced effects when parents view their own as opposed to other infants. Thus, when mothers view their own infants there is greater activation observed in many of the same brain areas discussed above associated with visual processing, emotion, empathy, theory of mind, reward processing, social cognition and motor control (Bartels and Zeki, 2004; Leibenluft et al., 2004; Nitschke et al., 2004; Noriuchi et al., 2008; Minagawa-Kawai et al., 2009; Laurent and Ablow, 2013; Stoeckel et al., 2014; Wan et al., 2014; Esposito et al., 2015).

There are also some additional brain areas activated when mothers view their babies, including visual processing regions such as the occipital and temporal cortices (BA 17/18/19/37) (Nitschke et al., 2004), the right anterior temporal pole (ATP—BA 38) (Ranote et al., 2004) involved in emotional processing, periaqueductal gray (PAG) (Bartels and Zeki, 2004; Noriuchi et al., 2008), lateral OFC and lateral prefrontal cortex (lPFC—BA 11/47/46/45) (Bartels and Zeki, 2004) involved in maternal responses and emotion/reward processing and anterior paracingulate cortex (APC—BA 9) (Leibenluft et al., 2004) involved in theory of mind and cognitive processing. Moreover, ERP studies have found that both amplitudes of the parietal-distributed P300, involved in attention, and the temporal N170 component implicated in face encoding, were increased during viewing of own infant/child but not of unfamiliar infants/children (Doi and Shinohara, 2012; Weisman et al., 2012c). These specific neural responses toward own infant/child faces may represent components of maternal love/attachment which are clearly very important for the socio-emotional and cognitive development of infants, especially during the early post-partum period.

One of the most consistent findings in terms of an own infant-specific neural response in mothers is increased activation in the OFC which plays a key role both in emotion and reward processing (Bartels and Zeki, 2004; Nitschke et al., 2004; Kringelbach et al., 2008; Stoeckel et al., 2014). The OFC includes medial (BA 25/14/10) and lateral (BA 47/12/11/10) areas and in accordance with its anatomy and connectivity the lateral portion has been further subdivided into another three sub-regions: anterior, posterior and caudal (Elliott et al., 2000). The medial portion is particularly involved in monitoring reward value and making stimulus-reward associations while the lateral portion is more related to stimulus-outcome associations and reward-related response suppression (Elliott et al., 2000; Walton et al., 2010). Overall, studies have found that maternal love (in terms of maternal responses to own babies) is mostly associated with increased activation of the lateral OFC. Thus bilateral lateral OFC (BA 11/47) activation occurs when mothers passively view images of their own as opposed to another familiar infant (Bartels and Zeki, 2004); happy faces of their own compared to another unfamiliar happy infant (Nitschke et al., 2004) or video clips of their own compared to unknown infants (Noriuchi et al., 2008; Minagawa-Kawai et al., 2009). Another study has also reported greater activation in the left inferior OFC (BA 11) with own vs. other joyful infant faces in mothers with low self-reported current depressive symptoms (Laurent and Ablow, 2013). Finally there is also a study showing increased activations in response to viewing an own infant in the anterior portion of the OFC (Minagawa-Kawai et al., 2009).

An increasing number of studies have investigated the neural substrates of paternal love in recent years (Atzil et al., 2012; Lamb and Lewis, 2013; Leidy et al., 2013; Mascaro et al., 2014). Neuroimaging studies on fathers viewing own infant/child faces have found increased activity in brain areas including medial and lateral frontal cortex (IFG, MFG, SFG, OFC), SMG, MTG, INS, cingulate, striatum (CAU) and AMY (Atzil et al., 2012; Kuo et al., 2012; Mascaro et al., 2014). All of these regions also show increased activation in mothers viewing their own infant/child faces. The only study to directly compare neural responses in mothers and fathers to videos of their own as opposed to other young infants also reported a considerable similarity between them (Atzil et al., 2012). However, this study also found significantly greater activation in mothers in a number of regions in the right hemisphere (STG, PostCG, FFG, MTG, AMY, lentiform nucleus, Cuneus, and CAU). On the other hand in fathers greater activation was found in the left MFG, inferior parietal gyrus, superior occipital gyrus and precuneus and in part of the right MTG and STG. This possibly suggests more right hemisphere dominated responses in mothers and left hemisphere dominated ones on father. Further evidence for differences between fathers and mothers has also been reported in response to own vs. other infant laughing of crying. In this case a deactivation response in the ACC, which is an important region in the control of emotion, was only found to occur in mothers (Seifritz et al., 2003). Somewhat surprisingly one study has reported that greater parental sensitivity and reciprocity in fathers was negatively associated with the activation in the right OFC for own compared with other infants (Kuo et al., 2012). This seems to imply that more responsive fathers may find all infants more rewarding, not just their own. However, only 10 subjects were included in this study and so some caution should be attached to interpreting this finding. Another a recent study has also investigated changes in regional gray matter (GM) volume in fathers from 2–4 to 12–16 weeks postpartum as an indication of potential neural plasticity changes. Results showed increased GM volume in the hypothalamus, AMY, striatum and lateral frontal cortex although a decrease in the OFC, PCC, and INS (Kim et al., 2014).

In summary, all the neuroimaging and electrophysiological findings described above support the conclusion that baby faces contain highly salient, affective and rewarding information that particularly engages the extensive neural processing systems involved in these functions in adult observers. These systems in turn mediate changes in motor preparation and response circuitry to promote approach, protection and nurturing behavior and the whole system undergoes plasticity changes to further strengthen the social bond with the infant and facilitate subsequent behavioral responses. Evidence to date suggests that the neural circuitry involved in maternal and paternal responses to own infants/children appears to be very similar, although there is some evidence for differential responses in some frontal, temporal, limbic, and brain reward regions. However further studies are clearly needed provide more extensive evidence for such parental sex differences.

## Hormonal correlates of attraction to infant faces

A number of cultural, experiential and physiological factors could potentially contribute to observed sex differences in responses to infant and child faces. In particular, differential responses in men and women as a result of cultural norms and expectations may play a significant role (Lytton and Romney, 1991), although this has not been systematically investigated in the context of the baby schema effect. However, there is also a growing amount of evidence for hormonal influences on responses to infant cues in terms of sex differences, effects of puberty, the menstrual cycle and pregnancy, sex hormones and also neuropeptides, such as oxytocin and vasopressin, involved in the control of parental behavior and social bonds.

There is increasing behavioral and neural evidence for sex differences in response to infant faces. Behavioral findings have shown that while both males and females find infant faces cute, females tend to be more sensitive to the cuteness of infant faces than males (Glocker et al., 2009a; Lehmann et al., 2013), have stronger reactions and attentional bias toward them (Seifritz et al., 2003; Cárdenas et al., 2013), exhibit a higher preference for and liking of them (Maestripieri and Pelka, 2002; Parsons et al., 2011; Charles et al., 2013) and make more effort and have a stronger motivation to view them (Hahn et al., 2013). However, it should be noted that one study has failed to find a gender difference in attraction to infant faces, although this might possibly reflect the young age of the male participants or possibly the rather limited 5-point Likert scale used (Sprengelmeyer et al., 2013).

This observed gender difference in the perceived attractiveness of infant faces may to some extent be attributed to increased responsiveness in brain reward regions in mothers compared to fathers (Atzil et al., 2012), and also to the larger size of the OFC in females relative to males (Gur et al., 2002; Proverbio et al., 2011b). Indeed, support for a bias in women finding infant faces more rewarding, rather than being more sensitive to recognizing them, comes from a study showing that females were only better than males at choosing which of a pair of infant faces was cuter, but not when deciding which was the younger or the happier one (Lobmaier et al., 2010).

Preferences for infant faces also vary in women across their life cycle, and particularly with regard to their reproductive status. Two studies have reported that while females overall exhibit an overall higher preference for infant faces than males this preference varies with age. The first of these studies reported that during childhood (6–10 years) and adolescence (11–15 years) females exhibited the highest preference, but that this declined thereafter during early (19–35 years) and later (46–75 years) adulthood. Interestingly males showed a relatively constant preference across all four age groups (Maestripieri and Pelka, 2002). A second study reported that young women (19–26 years) are more sensitive to infant cuteness than men aged 19–26 and 53–60 years old. Women aged 45–51 years were the same as younger women whereas those aged 53–60 years old showed a reduced cuteness sensitivity that was equivalent to men (Sprengelmeyer et al., 2009). Thus both studies suggest that female reproductive hormones may play an important role in increasing perceived cuteness of infant faces. This explains the sex difference between young women and men and the decline seen in older women who are likely to have undergone menopause.

Puberty and age of puberty have also been shown to influence perception of infant cuteness. Post-menarcheal girls have a higher preference for infant faces and rated them more positively than pre-menarcheal peers and boys, suggesting that the onset of menstruation may increase attention toward infantile features (Goldberg et al., 1982). Furthermore, girls who had an early menarche also exhibited a greater subsequent preference for infant faces than those who had a later onset menarche (Maestripieri, 2004; Maestripieri et al., 2004). This latter finding is perhaps a little surprising given that early puberty is generally more associated with a negative impact in terms of increased likelihood of depression and behavioral problems (Copeland et al., 2010). However, it was also found that both early menarche and increased perception of cuteness were associated with early paternal absence from the home and so it is argued that this may represent an adaptation in terms of an earlier readiness for reproduction and parenting (Maestripieri et al., 2004).

Two studies to date have investigated potential differences in responses to infant faces across the menstrual cycle. One of these did not find any effects (Sprengelmeyer et al., 2013), however the other using a forced-choice paradigm where subjects indicated which of two infant faces was cuter, found that women were more likely to choose the cuter baby during their ovulatory than luteal phase of the cycle (Lobmaier et al., 2015). Nevertheless, cuteness discrimination was not associated with saliva concentrations of oestradiol, progesterone or testosterone, leading the authors to speculate that this menstrual cycle phase effect might be associated with other relevant hormones which change during the cycle, such as oxytocin or prolactin. To date no studies have looked at changes across pregnancy. However, overall findings do suggest some potential links between female reproductive hormones and their sensitivity to the cuteness of infant faces and that this can therefore contribute to facilitation of parental caregiving in individuals who have the reproductive potential to produce children (Sprengelmeyer et al., 2010).

In fathers two studies have reported correlations between blood testosterone concentrations and neural responses to infant cues. Testosterone concentrations were found to be decreased in fathers compared to non-fathers, and negatively associated with activation of the MFG in response to pictures of young children (Mascaro et al., 2014). On the other hand a study has also reported a positive association between testosterone concentrations in fathers and activation of a brain reward area, the left CAU, following interaction with an infant (Kuo et al., 2012). Thus the relationship between testosterone and neural responses to infant cues in fathers is somewhat unclear.

## Effects of sex hormones on attraction to infant faces

While no studies have systematically investigated the effects of exogenous treatments with either estradiol or progesterone on sensitivity to cuteness in infant faces one has reported that women using oral contraceptives (which contain estrogen and progesterone) are more sensitive compared to those who do not (Sprengelmeyer et al., 2009). However, the same group in a subsequent study failed to find an effect of oral contraceptives on the aesthetic and incentive salience of cute infant faces (Sprengelmeyer et al., 2013).

Another important sex hormone which influences parenting behaviors is testosterone (Bos et al., 2010; Kuo et al., 2012). Testosterone may play a role in regulating females' reward sensitivity since it has been shown to increase the reward value of financial incentives through testosterone administration (Hermans et al., 2010). While no studies to date have investigated effects of testosterone administration on viewing baby faces several have attempted to find any associations with salivary testosterone concentrations. Thus higher salivary testosterone concentrations in women were associated with greater reward scores given to cute infant faces and this effect was independent of progesterone and estradiol concentrations (Hahn et al., 2014). Higher salivary testosterone concentrations in fathers during interactions with infants have also been associated with greater activation in brain reward regions such as the CAU when processing own vs. other infant faces (Kuo et al., 2012).

### Effects of oxytocin and vasopressin on attraction to infant faces

There has been considerable interest in the role of the evolutionary conserved hypothalamic neuropeptides oxytocin and vasopressin in recent years and a large number of studies have investigated their importance for a wide range of human social and emotional behaviors. The majority of studies have focused on oxytocin (OXT) and its effects on trust, cooperation, face emotion recognition, empathy, in-group preferences and also on social bonds and maternal attachment (Bartz et al., 2011; Kemp and Guastella, 2011; Striepens et al., 2011; Weisman et al., 2012a,b; Scheele et al., 2014; Wigton et al., 2015). Oxytocin may play an important role in human parental responses, with higher plasma concentrations of maternal oxytocin across pregnancy being predictive of higher quality of postpartum maternal care (Feldman et al., 2007). Increased plasma concentrations of oxytocin across the first 6 months following the birth of a child have also been correlated with various, although differing, positive aspects of parental responsiveness in both mothers and fathers (Gordon et al., 2010). Associations between oxytocin receptor polymorphisms (Riem et al., 2011b; Feldman et al., 2012) and the vasopressin V1a receptor (Bisceglia et al., 2012) and sensitive parenting have also been reported. Oxytocin released during breast-feeding may also have stress-reducing effects (Heinrichs et al., 2001, 2002). In the context of the current review associations between oxytocin and vasopressin and attraction to infant faces have been shown by demonstrating correlations between plasma or saliva concentrations or associations with receptor polymorphisms or neural and behavioral responses to exogenous treatment using intranasal application.

Salivary oxytocin concentrations have been found to be positively correlated to mood ratings of happy but not sad infant faces in women (Bhandari et al., 2014). In an ERP study, urinary oxytocin concentrations in foster mothers following a cuddle interaction with their infants were also shown to be positively correlated with P300 amplitude in response to viewing all infant faces (Bick et al., 2013). However, another recent study which failed to demonstrate differences in responses to infant compared with adult faces in a facial visual research task found that urinary oxytocin concentrations were positively correlated with performance on both types of faces (Saito et al., 2014). Higher concentrations of plasma oxytocin have also been found to be related with stronger maternal response in terms of increased gaze toward the infant face in postpartum mothers (Feldman et al., 2007; Gordon et al., 2010), and greater activation in brain reward regions such as the ventral striatum, OFC and medial frontal cortex as well as in hypothalamic/pituitary regions in first-time mothers viewing own vs. unknown infant faces (Strathearn et al., 2009). Furthermore, those mothers with lower plasma oxytocin concentrations when viewing their own vs. unknown infant faces were found to be more likely to have insecure attachment and also reduced activation of the mesocorticolimbic dopamine reward system in response to infant face cues (Strathearn et al., 2008, 2009; Strathearn, 2011).

Thus while plasma, salivary and urinary concentrations of oxytocin may not necessarily always accurately reflect those in the brain (see Striepens et al., 2011) there does seem to be some association between higher endogenous concentrations of the peptide and enhanced responses to infant face cues, at least in post-partum mothers. In line with the potential role of oxytocin in influencing social reward and modulating activity in brain reward systems (Scheele et al., 2013; Striepens et al., 2014), increased peripheral concentrations also seem to be associated with greater responses to own vs. other infant faces and cues in dopaminergic reward pathways (Rilling, 2009; Strathearn et al., 2009; Strathearn, 2011). However, this relationship between oxytocin and enhanced activation in brain reward systems is not specific to parent-infant bonds since it has also been reported for romantic bonds in terms of men viewing the face of their female partner compared with another either familiar or unfamiliar woman (Scheele et al., 2013).

Intranasal oxytocin administration has been found to enhance responses to important infant cues such as crying (Riem et al., 2011a, 2014) and laughing (Riem et al., 2012). Oxytocin administration has also been reported to increase preference for infant faces in homozygous GG allele carriers for the rs53576 polymorphism of the oxytocin receptor, whereas rs53576A allele carriers showed the opposite pattern (Marsh et al., 2012). An ERP study has linked the rs53576 polymorphism with sensitivity to infant cues since in both mothers and nulliparous women who were GG allele carriers an early (~ 100 ms) differential frontal ERP response to strong intensity infant face expressions was associated with faster emotion recognition performance (Peltola et al., 2014). The same study found that mothers exhibited modulation of the early posterior negativity component (EPN) by negative valence faces. Intranasal OXT administration has also been shown to enhance subjective arousal ratings for infant photos in nulliparous women, and their ratings were positively correlated with their AMY activation in the oxytocin but not placebo treatment group (Rupp et al., 2013). Another study using the Infant Facial Expressions of Emotions from Looking at Pictures (IFEEL) task showed that oxytocin increased activation in empathy-related brain regions such as the left inferior frontal gyrus (IFG), MTG, and STG when women judged the emotion vs. gender of the infant faces. However, surprisingly it decreased behavioral performance on the face emotion recognition task independent of the difficulty level (Voorthuis et al., 2014). Oxytocin has also been reported to increase activity in the VTA, but not accumbens, of both nulliparous women and post-partum mothers during viewing of infant faces (Gregory et al., 2015). Studies investigating both neural and behavioral effects of intranasal oxytocin on responses to infant faces are summarized in Table 3.

**Table 3:** **The effect of oxytocin administration on brain response to infant and child faces**.

**Study**	**Participants**	**Face age**	**OXT dose**	**Face presentation**	**Behavior task**	**Contrasts**	**WB/ROI**	**Neural effect of OXT**
Voorthuis et al., 2014	50 nulliparous women: 18–27 years	N/A	16 IU	5 s	Adapted version of the Infant Facial Expressions of Emotions from Looking at Pictures (IFEEL pictures) task: to indicate the child's emotional state or the gender	Emotion > gender judgment	ROI ROI	↑L STG ↑L MTG
Wittfoth-Schardt et al., 2012	21 Fathers: 39.3 ± 6.2 years	3–6 years	24 IU	2 s	Implicit facial processing	Own > familiar child	ROI	↓ L GP
						Unfamiliar > familiar child	ROI WB ROI WB WB WB	↓ L GP (PUT) ↓ L preCG ↓ L HIPP (AMY) ↓ L/R MTG ↓ L STG ↓ L SMG (IPS)
						Own > unfamiliar child	WB	↑ L CAU
						Own > familiar child (functional connectivity)	ROI/WB ROI/WB ROI/WB ROI/WB	↓ L GP, R GP ↓ L GP, L MFG ↓ L GP, L HIPP ↓ L GP, R SPL
Gregory et al., 2015	30 nulliparous female; 29 postpartum female (16 primiparous, 13 multiparous); 20-40 years	N/A	24 IU	2s	One-back matching task: A sexually explicit, crying infant, smiling infant and neutral photos.	Crying infant > fixation	ROI	↓VTA

*OXT, oxytocin; IU, international unit; WB, whole brain analysis; ROI, regions of interest analysis; L, left; R, right; ↑, increased; ↓, decreased. Abbreviations of brain regions: see Table 2*.

While fewer studies have been carried out on men/fathers, one has reported the intranasal oxytocin treatment actually reduced activation in reward- and attachment-related brain regions, such as the left GP, when biological fathers passively viewed their own vs. an unfamiliar child (3–6 years) or an unfamiliar vs. familiar child. Oxytocin also decreased functional connectivity within a fronto-pallido-hippocampal network for own vs. unfamiliar child (Wittfoth-Schardt et al., 2012). Therefore, oxytocin may have differential effects on mothers and fathers by selectively modulating functional brain responses and connectivity to infant faces in regions associated with emotion, attachment, novelty and reward processing (Wittfoth-Schardt et al., 2012). Another study has also reported different associations between plasma oxytocin concentrations in mothers and fathers and brain regions showing greater responses to videos of own vs. other infants (Atzil et al., 2012). Thus, while higher AMY activation in mothers was positively associated with plasma oxytocin concentrations, this was not the case in fathers. In mothers oxytocin concentrations were positively associated with activity in the left INS, left inferior parietal lobule (IPL), bilateral temporal cortex (TC), left ventral ACC and left NAcc. In fathers on the other hand activation in the left IFG, SFG and medial prefrontal cortex (mPFC), left postCG and left ACC was negatively associated with oxytocin concentrations. These findings again support the possibility that oxytocin may be influencing brain regions associated with attention, emotion, reward and even motor processing differently in mothers and fathers, although obviously some caution needs to be applied to such simple correlational analyses of this kind.

A number of studies have reported effects of intranasal oxytocin on reducing AMY responses to negative emotional faces (see Striepens et al., 2011) and also to both laughing (Riem et al., 2012) and crying (Riem et al., 2011a) infants. However despite the fact that greater AMY activation has been reported in response to own infant faces in mothers (Atzil et al., 2012; Strathearn and Kim, 2013), effects of oxytocin on AMY responses to infant faces have so far not been found.

Vasopressin, which is closely associated with oxytocin, has also been shown to influence social behaviors (Hammock, 2015; Patel et al., 2015). In rats, for example, it plays a potent role in facilitating maternal behavior, independent of trait anxiety (Bosch and Neumann, 2008). However, to date few studies have investigated potential effects of vasopressin on the attractiveness of infant cues. One study has reported some overlapping but also different patterns of negative associations between plasma vasopressin concentrations and activity in brain regions responding more strongly to videos of own vs. other infants (Atzil et al., 2012). In mothers, associations were found in bilateral SFG, right MFG and right middle temporal gyrus (MTG), whereas in fathers they were found in the right IPL, right inferior and medial frontal gyri, left INS and right temporal lobe. Thus, as with oxytocin, there may be different responses to vasopressin in maternal and paternal brains, although this clearly needs more detailed confirmation. While at this stage it is unclear whether vasopressin may play an important role in influencing responses to infant cues by either males or females, one speculation might be that it could serve to enhance empathic responses, particularly in those individuals whose parental response sensitivity is high. For example, a recent study has reported that intranasal vasopressin, but not oxytocin, increased empathic concern in both male and female subjects. Interestingly this effect was strongest in individuals who had received higher levels of paternal warmth during their childhood (Tabak et al., 2014).

## Altered responses to infant cues in post-partum depression and substance abuse

Post-partum depression affects between 6.5 and 8.5% of mothers (Yonkers et al., 2001) and is associated with reduced positive interest and responses to infant cues, which in turn can lead to weakening the relationship between a mother and her child. Studies have shown that mothers suffering from postnatal depression are more likely to rate negative emotion infant faces more negatively (Stein et al., 2010), and even neutral expression ones more negative than controls (Gil et al., 2011). Mothers with post-partum depression are also less accurate when identifying unfamiliar happy infant faces than healthy mothers, although there were no differences found when identifying sad faces (Arteche et al., 2011). While paternal postpartum depression is also moderately and positively correlated with maternal depression (Paulson and Bazemore, 2010), no study to date has investigated altered responses of fathers with depression to infant cues. This should be an important area for future studies.

Neuroimaging and electrophysiological studies have found evidence for a reduced effect of infant faces in a number of the same brain regions involved in attention, emotional and empathic responses and reward discussed above. Thus, depressed mothers compared to healthy controls showed a slower response in the dorsal ACC when viewing the distressed face of their own infant. Also, those with higher levels of current symptomatology showed reduced responses in the OFC and INS toward their own infant's joyful faces. Symptom severity could also predict lower responses to their own infant in left prefrontal and insula/striatal regions (Laurent and Ablow, 2013). On the other hand an ERP study has reported that the face-sensitive N170 component elicited in response to infant face stimuli was positively related with depression symptom severity (Noll et al., 2012). This perhaps implies an increased initial automatic perceptual sensitivity to infant faces in mothers with a greater severity of post-partum depression, but a subsequent suppression of responses in brain regions controlling positive attentional, emotional and reward responses to infants.

Anxiety disorders can also impact negatively on maternal responses to infants and one study has shown that mothers with generalized anxiety disorder are inclined to rate the intensity of happy infant faces lower than controls (Arteche et al., 2011). Furthermore, the babies of anxious mothers appear to be less willing to look at their face since maternal anxiety scores have been shown to be negatively correlated with the amount of time babies looking at their mother's face (Jones et al., 2013). Thus babies also appear to be sensitive to reduced interest in them by anxious, and probably also depressed mothers, thereby further increasing the potential threat to the parent-infant bond.

Key regions exhibiting altered responses to negative emotional stimuli in patients with anxiety and depression disorders notably include those involved in responses to infant faces, such as the ACC, INS, and AMY (Jaworska et al., 2014; Oathes et al., 2015).

Drug addiction has also been shown to influence responses to infant faces. The National Survey on Drug Use and Health (NSDUH) in 2007 found that 5.2% of pregnant women reported using illicit drugs during pregnancy; and an additional 11.6% reported using alcohol and 16.4% tobacco. Mothers using cocaine during pregnancy have been found to respond more passively and in a more disengaged way to their babies (Gottwald and Thurman, 1994), and similar patterns of reduced responsivity in substance-using mothers have been reported in terms of parenting children even beyond infancy (Johnson et al., 2002; Molitor and Mayes, 2010). An fMRI study has shown that mothers using drugs during pregnancy (tobacco, heroin, marijuana, opiates, cocaine, and alcohol) had reduced responses to neutral and emotional infant faces in many of the regions discussed above which show enhanced responses to infant faces. For happy faces reduced responses were found in frontal regions involved in attention, salience and reward (ventromedial, dorsolateral and dorsomedial frontal cortex) as well as in early visual processing (occipital gyrus). For sad faces similar reductions were seen in frontal regions (dorsolateral frontal cortex, inferior and medial frontal gyri and medial OFC), although additionally in sensorimotor regions, MTG, STG, and PCC, as well as the AMY and parahippocampal gyrus (PHG). For neutral expression faces again there were extensive frontal reductions in responses (ventromedial, dorsomedial, and dorsolateral frontal cortex and inferior frontal gyrus) as well as in sensorimotor regions, PCC, GP, AMY, and PHG. There was also reduced responsiveness a primary visual processing region, the cuneus (Landi et al., 2011). Thus overall, drug taking appears to have an even more pronounced effect in reducing responsiveness in brain circuitry to infant faces than either post-partum depression or general anxiety.

## Conclusions and future directions

In line with the potent impact of facial baby schema on adult attraction, protection and caregiving behaviors, neuroimaging and electrophysiological studies reveal an extensive neural circuitry involved in our perception of infant faces. Enhanced activation in response to infant compared to adult faces is found in cortical and sub-cortical brain regions involved in face perception, attention, emotion, empathy, memory, reward and attachment, theory of mind and also control of motor responses. Both mothers and fathers also show evidence for enhanced responses in these same neural systems when viewing their own as opposed to another child. Importantly post-partum depression, anxiety and drug-taking all tend to reduce responsivity in this neural circuitry involved in processing and responding to infant face cues, with the most extensive changes in this respect appearing to occur in women taking addictive drugs during pregnancy.

Reproductively active women tend to rate infant faces as cuter than men and this may be mainly a reflection of both heightened attention to relevant cues and a stronger activation in their brain reward circuitry. In both sexes perceived cuteness of infant faces is influenced by reproductive hormones, with women in particular showing an ovulatory peak in interest during their cycle and an apparent decline post-menopause. To date evidence does not support major roles for the gonadal hormones estradiol, progesterone and testosterone in influencing responsivity to infant faces, although there is increasing evidence linking oxytocin with facilitation of attention toward and attractiveness of infant cues in both sexes.

Future studies need to explore in more detail the functional relevance of specific components of the widespread neural circuitry associated with the enhanced responses to infant faces. To date, for example, only one study has demonstrated the functional importance of the dorsolateral prefrontal cortex in the processing of emotional baby faces using rTMS (Baeken et al., 2010b). A particular focus should be on the circuitry involved with face processing, attention, emotion, empathy and reward processing since this would appear to be affected in reduced responses observed in post-partum depression, anxiety and drug use. It is also important to establish the functional roles of the neuropeptides oxytocin and vasopressin in mediating enhanced neural and behavioral responses to infant faces and other salient cues since they could in future represent potential therapeutic agents.

### Conflict of interest statement

The authors declare that the research was conducted in the absence of any commercial or financial relationships that could be construed as a potential conflict of interest.
